# QuantiFERON-TB Gold Plus Assay in Patients With Latent vs. Active Tuberculosis in a Low Incidence Setting: Level of IFN-γ, CD4/CD8 Responses, and Release of IL-2, IP-10, and MIG

**DOI:** 10.3389/fmicb.2022.825021

**Published:** 2022-04-08

**Authors:** Séverine Carrère-Kremer, Pratt Kolia-Diafouka, Amandine Pisoni, Karine Bolloré, Marianne Peries, Sylvain Godreuil, Arnaud Bourdin, Philippe Van de Perre, Edouard Tuaillon

**Affiliations:** ^1^Pathogenesis and Control of Chronic and Emerging Infections, University of Montpellier, INSERM U1058, EFS, Antilles University, Montpellier University Hospital, Montpellier, France; ^2^UMR MIVEGEC IRD-Centre National pour la Recherche Scientifique (CNRS), University of Montpellier, Montpellier University Hospital, Montpellier, France; ^3^PhyMedExp, INSERM U1046, Centre National pour la Recherche Scientifique (CNRS) UMR 9214, University of Montpellier, Montpellier University Hospital, Montpellier, France

**Keywords:** tuberculosis, QuantiFERON TB Gold plus^®^, IL-2, IP-10 (CXCL-10), latent tuberculosis, active tuberculosis, cytokines, interferon-gamma (IFN-γ)

## Abstract

**Objectives:**

We analyzed the results of the QuantiFERON Glod Plus assay (QFT) and cytokine patterns associated with active tuberculosis (ATB) among patients with positive QFT.

**Methods:**

A total of 195 patients are QFT-positive, among which 24 had an ATB and 171 had a latent tuberculosis infection (LTBI). Interferon-gamma (IFN-γ) secretion was analyzed relative to interleukin-2 (IL-2), IFN-γ inducible protein or CXCL-10 (IP-10), and monokine induced by IFN-γ or CXCL-9 (MIG) secretion, and then compared between two sets of peptide antigens [tube 1 - cluster of differentiation 4 (CD4^+^) T cell stimulation; tube 2 - CD4^+^/CD8^+^ T cell response].

**Results:**

Higher IFN-γ responses were measured in the ATB group (*p* = 0.0089). The results showed that there was a lower ratio of tube 1/tube 2 IFN-γ concentrations in the ATB group (*p* = 0.0009), and a median [interquartile ranges (IQR)] difference between the two sets at −0.82 IU/ml (−1.67 to 0.18) vs. −0.07 IU/ml (−0.035 to 0.11, *p* < 0.0001) in the ATB group compared to the LTBI group, respectively. In addition, patients with low ratios of IL-2/IFN-γ, IP-10/IFN-γ, and MIG/IFN-γ were much more likely to have ATB.

**Conclusion:**

High levels of IFN-γ secretion, preferential IFN-γ response in tube 2, and lower secretion of IL-2, IP-10, and MIG release relative to IFN-γ secretion were more likely observed in subjects with ATB. These features of T cell response may be helpful in low prevalence settings to suspect ATB in patients tested positive for IFN-γ release assays (IGRA).

## Introduction

Tuberculosis (TB) remains a major health problem worldwide with 1.5 million deaths per year (World Health Organization [WHO], 2021). Latent TB infections (LTBI) are defined as a state of the persistent immune response against *Mycobacterium tuberculosis* (*Mtb)* without clinically manifested evidence of active TB (ATB). Out of the 1.7 billion people with latent tuberculosis (LTBI), it is estimated 5–15% will progress to ATB (Vynnycky and Fine, 2000). Interferon-gamma (IFN-γ) release assays (IGRAs) have been introduced as an alternative to the tuberculin skin test (TST) to detect the immune response against *Mtb* while avoiding *in vivo* screening tests, cross-reactions with bacille Calmette-Guérin (BCG) vaccination, and non-tuberculous mycobacterial infections (Huebner et al., 1993). Hence IGRAs are frequently preferred to TST in routine practice. The QuantiFERON assay (QFT, Qiagen) quantifies IFN-γ released after incubation of whole blood with a cocktail of peptides derived from 6-kDa Early Secretory Antigenic Target (ESAT-6) (Rv3875) and 10-kDa Culture Filtrate Antigen (CFP-10) (Rv3874) antigens. This assay is increasingly used in Europe for the screening of health care workers and for the diagnosis of those with LTBI who are at risk for progression to ATB, including persons living with HIV, persons recently exposed to ATB, children, and persons who had prior anti-TNF therapy. One of the major restrictions of IGRA is its inability to distinguish between ATB and LTBI. TB diagnosis still remains a major concern having important public health implications. The incidence of ATB is low in West European countries but the prevalence of LTBI remains significant. LTBI prevalence has been recently estimated at 6.3% in France in the general population, i.e., 4,158,000 persons (Houben and Dodd, 2016), of which 15% are health care workers exposed to ATB (Lucet et al., 2015). In comparison, 4,741 ATB were notified in 2015 in France, i.e., 7.1 per 100,000 habitants (Guthmann et al., 2017). Even when the level of clinical suspicion for TB is low, it is sometimes challenging to rule out ATB in patients tested positive for IGRA. In other words, it is a question of how to identify patients needing TB culture and molecular tests when IGRA is positive.

Several studies suggested that the accuracy of IGRA in discriminating ATB vs. LTBI disease can be improved by parallel assessment of the profile of *Mtb*-specific T cells for other cytokines secreted along with IFN-γ. Interleukin-2 (IL-2) was the most extensively investigated alternative immunological biomarker (Hur et al., 2015; Wergeland et al., 2016; Won et al., 2017; La Manna et al., 2018; Lesosky et al., 2019). IFN-γ inducible protein or CXCL-10 (IP-10) (Jeong et al., 2015; Wergeland et al., 2016; Villar-Hernández et al., 2017) and monokine induced by IFN-γ or CXCL-9 (MIG) (Frahm et al., 2011; Carrère-Kremer et al., 2016) have also been highly analyzed. The pattern of T helper cell type 1 (Th1)-pro-inflammatory cytokines could improve the sensitivity for ATB diagnosis alone (Biselli et al., 2010; Borgström et al., 2012; Biraro et al., 2016) or combined with IGRA results (Suter-Riniker et al., 2011; Carrère-Kremer et al., 2016), but the conclusions of the studies were sometimes conflicting. Authors have also suggested that a strong IFN-γ response of cluster of differentiation 8 (CD8^+^) T cells stimulated by ESAT-6 and CFP-10 may be associated with ATB (Nikolova et al., 2013; Rozot et al., 2013). Qiagen company launched the latest generation of a QFT assay in 2015 (QuantiFERON-TB Gold Plus) including an additional new set of peptides designed to elicit both CD8^+^ and CD4^+^ T cell responses (Collins and Kaufmann, 2001; Day et al., 2011).

We previously reported that the detection of a high number of IFN-γ secreting cells using T-SPOT test and an impaired capacity of multi-cytokine release measured by a multiplex microbeads-based method had both appeared as signatures of ATB in a low prevalence setting (Carrère-Kremer et al., 2016). In this study, we assessed the IL-2, IP-10, and MIG response relative to IFN-γ secretion in patients with ATB and LTBI. The IFN-γ secretion in QFT Plus tube 1 containing peptides, which was designed to stimulate CD4^+^ T cell response, was also compared to tube 2 peptides, which was designed to elicit both CD4^+^/CD8^+^ T cell response.

## Materials and Methods

### Patients and Samples

This case-control study was conducted in the Montpellier University Hospital (France) in outpatients and hospitalized adults. Subjects were enrolled based on clinical presentation and their positive IGRA result. Samples were collected at diagnosis, before initiation of any tuberculosis treatment, or no later than 7 days after tuberculosis diagnosis. People living with HIV and immunocompromised subjects were excluded from the study. The status ATB vs. LTBI was established on *Mtb* culture results, CT scan, and after multidisciplinary chart review based on clinical presentations and outcomes. The study was performed in accordance with the guidelines of the Helsinki Declaration and was approved by the local ethics committee (Sud-Méditerrannée-III, France, NCT02898623).

### Laboratory Methods

#### QuantiFERON-Tb Gold Plus Assay

The assay was performed according to the manufacturer’s instructions (Qiagen, Darmstadt, Germany). Briefly, 1 ml of whole blood was drawn into the three QFT tubes coated with saline (Nil Control), which were peptide cocktails simulating ESAT-6, CFP-10 (*Mtb* antigens), or phytohemagglutinin (Mitogen Control), and incubated at 37°C. Following 20 h incubation, the plasma was harvested from each tube to determine the IFN-γ concentration. The highest value between the QTF tube 1 (TB1) and tube 2 (TB2) was retained for comparison between LTBI and ATB groups.

#### ELISA

Interleukin-2, IP-10, and MIG secretion were quantitated in supernatants of QFT Plus tube 1 and negative control tube (Nil) using Duoset ELISA Development kits for (Bio-Techne, MN, United States). The supernatant was diluted at 1:2 for all cytokines except 1:10 for IP-10 measurement. Levels were obtained by subtracting the Nil from the TB1 levels. Results were expressed in pg/ml.

### Statistical Analysis

Results were analyzed using GraphPad Prism 6 (GraphPad Software, La Jolla, CA, United States) and SAS 9.4 (SAS/STAT Software, Cary, NC, United States). The median concentration of IFN-γ in paired TB1 and TB2 was compared using a Wilcoxon signed-rank test. The median cytokine levels [interquartile ranges (IQR)] of the ATB and LTBI groups were compared using the non-parametric Wilcoxon Mann–Whitney test. A *p*-value < 0.05 was considered significant. The receiver operating characteristics (ROC) curves were constructed by plotting the true positive rate (ATB samples; sensitivity) against the false positive (LTBI samples; 1-specificity). Areas under the curve (AUCs) were calculated along with their 95% CIs. Cut-offs for cytokines were determined using the Youden index, which was defined as sensitivity + specificity-1, and a visual appreciation on scatter plots. With these cut-offs, crude and adjusted odds ratios (ORs) were calculated using logistic regression. Multivariate analysis was not performed since parameters were all generated based on IFN-γ secretion (IFN-γ secretion, cytokines/IFN-γ ratios, and IFN-γ in TB1 vs. TB2).

## Results

### Patients Characteristics

A total of 195 patients who tested positive with QFT were enrolled, among which 24 (12%) had an ATB and 171 (88%) had an LTBI. Clinical characteristics of the patients are shown in Table 1.

**TABLE 1:** Patients’ characteristics.

Characteristic	ATB	LTBI
Numbers of patients	24*	171
Median age in year (IQR)	38 (29–58)	47 (32–62)
Number of females (%)	10 (42%)	92 (53%)
IFN-g [IU/ml, median (IQR)]	5.34 (1.15–8.9)	1.24 (0.69–4.1)
Smear positive	5	0
Culture positive	24	0

*TB localization: pulmonary localization (n = 15); pulmonary + extra pulmonary (n = 8); 1 extra pulmonary.

### A High Value of IFN-γ Is Associated With an Increased Risk of Active Tuberculosis

**FIGURE 1:**
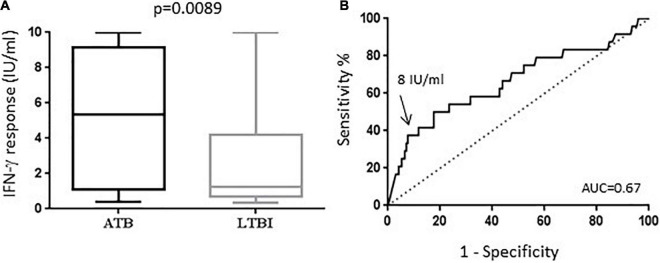
QFT results for discrimination between active and latent tuberculosis. **(A)** The level of IFN-γ in ESAT-6, CFP-10 simulated T cells was quantified by the QFT assay from blood samples of 24 patients with ATB (black boxplot) and 171 patients with LTBI (gray boxplot). The median, IQR, 10th and 90th is shown in boxplots of each group. The *p*-value was calculated by the Mann–Whiteny U test. **(B)** The ROC curve shows sensitivity vs. specificity for IFN-γ in differentiating the ATB from LTBI group. The AUC and a threshold at 8 IU/ml are indicated in the graph.

Higher levels of IFN-γ secretion were measured in the ATB group, median (IQR): 5.34 IU/ml (1.09–9.15) compared to patients with LTBI 1.24 IU/ml (0.69–4.21), *p* = 0.0089 (Figure 1A). ROC curves were established to evaluate the capacity of the IFN-γ secretion in order to distinguish ATB and LTBI in our population (Figure 1B). The AUC of IFN-γ secretion was 0.67 (95% CI 0.53–0.79). Based on the Youden index, we determined an optimal cut-off value at 8 IU/ml corresponding to a specificity of 92% and a sensitivity of 38%. By selecting patients with a QFT value greater than 8 IU/ml, 9 out of 24 (37.5%) from the ATB group and 13 out of 171 (7.6%) from the LTBI group had results above this cut-off, which led to OR = 7.8 for ATB (Table 2).

**TABLE 2:** Concentrations in pg/ml of cytokines secreted by T cells in QuantiFERON (QFT) supernatants after ESAT-6, CFP-10 stimulation in 24 subjects with active tuberculosis (ATB) and 171 with latent TB infection (LTBI).

	ATB pg/ml [median (IQR)]	LTBI pg/ml [median (IQR)]	*p*-value
IL-2	163 (33–456)	85 (10–290)	*p* = 0.128
IP-10	2907 (997–6955)	4416 (2000–6849)	*p* = 0.631
MIG	844 (379–1524)	948 (267–1806)	*p* = 0.810

*LOD, the limit of detection; n, number of values above detection level; nsd, non-significantly different. ATB, active tuberculosis; LTBI, latent tuberculosis infection; IQR, interquartile range.*

### A Stronger Response in the Second QTF Antigen Tube Compared to the First Tube Is Associated With Active Tuberculosis

**FIGURE 2:**
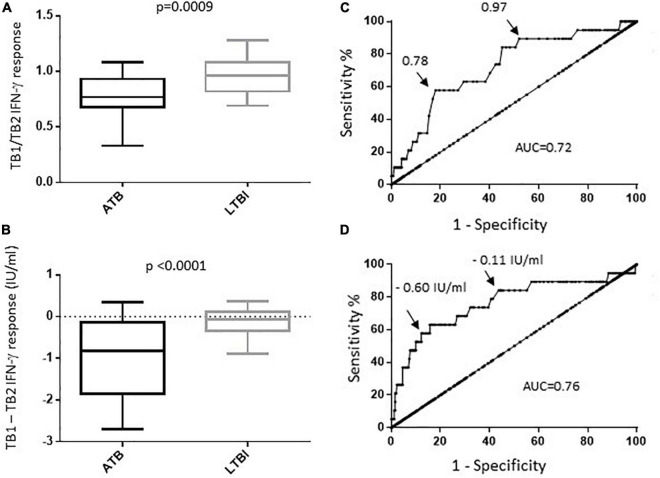
Comparison of IFN-γ response in QTF tube 1 versus tube 2. Tube 1 (TB1) contain ESAT6 and CFP10 peptides designed to stimulated TB specific CD4^+^ and CD8^+^ T cell mediated responses. **(A)** Ratio of TB1/TB2 IFN-γ response. The median, IQR, 10th and 90th are shown in boxplots for each group. **(B)** Difference of the IFN-γ response in TB1 minus TB2 in ATB and LTBI. The median, IQR, 10th and 90th are shown in boxplots for each group. **(C)** ROC curve for TB1/TB2 IFN-γ response in differentiating ATB and LTBI. **(D)** ROC curve for TB1 minus TB2 IFN-γ response in differentiating ATB and LTBI. The AUC and tradeoff values are indicated on the ROC curves.

The concentrations of IFN-γ in supernatants were compared after CD4^+^ peptides stimulation (TB1) and CD4^+^/CD8^+^ peptides stimulation (TB2). The contribution of CD8^+^ T-cell to IFN-γ secretion was first expressed as a ratio of IFN-γ TB1/TB2 concentration (Figure 2A). A lower ratio was observed in the ATB group compared to the LTBI group [median (IQR):0.77 (0.69–0.91) vs. 0.96, (0.82–1.08; *p* = 0.0009), respectively]. The difference was also estimated by subtracting the quantitative value of the TB2 to TB1 (Figure 2B). We observed the largest difference between the two sets of peptide antigens in the ATB group compared to LTBI, with a median (IQR) difference of −0.82 IU/ml (−1.67 to 9.18), vs. −0.07 IU/ml (−0.035 to 0.11; *p* < 0.0001), respectively.

### Cytokine Association Highlights Active Tuberculosis Risk Groups

QuantiFERON supernatants from the tube containing antigens dedicated to CD4^+^ T cell stimulation (TB1) were tested by ELISA. IL-2, IP-10, and MIG concentrations were not found significantly different between the two groups (Table 2).

**FIGURE 3:**
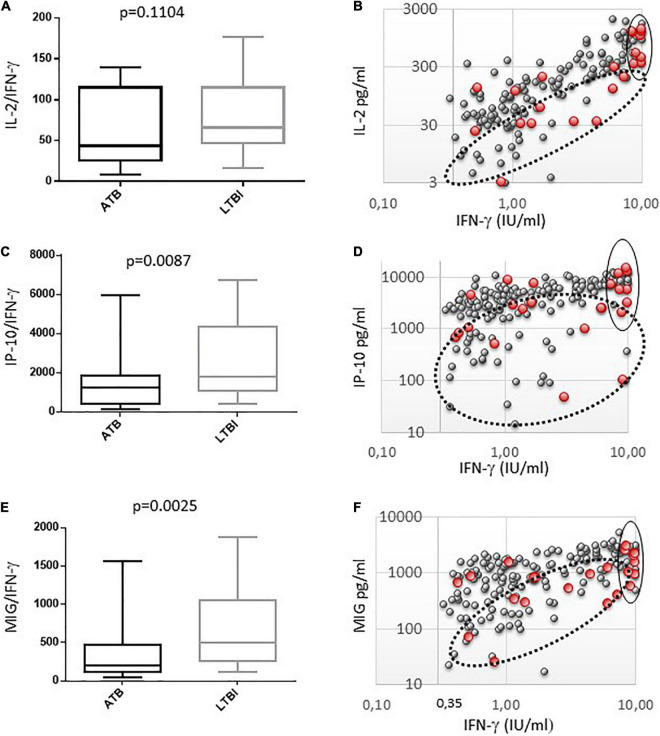
Comparison of cytokine response in QFT supernatant between active (ATB) and latent TB infections (LTBI). **(A,C,E)** IL2, IP10 and MIG indexes (cytokine/IFN-γ) are represented, ATB (black boxplots) and LTBI (gray boxplots). The median, IQR, 10th and 90th are shown in boxplots for each group. *p*-values are calculated with the Mann–Whiteny U test. **(B,D,F)** Biparametric graphs between the levels of IFN-γ (IU/ml) and cytokines (pg/ml) in QFT supernatants. ATB are represented by black circles and LTBI by gray circles. Risk groups are circled, subjects with ATB had more tend to have lower ratios of IL-2/IFN-γ, IP-10/IFN-γ, and MIG/IFN-γ (doted line circles), and high IFN-γ response (solide line circles).

The ratios of IL-2, IP-10, and MIG to IFN-γ were significantly lower in the ATB group than in LTBI group (Figures 3A,C,E). The indices for ATB and LTBI were respectively 43.6 (26.5–110.3) vs. 66.7 (47.3–115.1) for IL-2 (*p* = 0.091), 1298.6 (572.8–1863.5) vs. 4383.8 (1065.7–4326.7) for IP-10 (*p* = 0.00932), and 201 (112–476) vs. 502 (264–1052) for MIG (*p* = 0.00288).

Using ROC curves, AUC were 0.63, 0.63, and 0.66 for IL-2/IFN-γ, IP-10/IFN-γ, and MIG/IFN-γ ratios, respectively (data not shown). Thresholds were determined using Youden indices to determine ATB risk groups with ORs (Figure 2C,D,Table 3).

**TABLE 3:** Crude odds ratios identifying associations between ATB and interferon-gamma (IFN-γ) from ESAT-6, CFP-10 stimulated whole blood (QFT).

Cytokines (threshold value)	OR	95% CI	*p*-value
IFN-γ (≥ 8*)	7.2	2.6–19.8	0.0001
IFN-γ TB1/TB2 (≤ 0.78)	6.6	2.5–17.2	0.0001
IFN-γ TB1–TB2 (≤ −0.60*)	8.3	3.1–22.0	<0.0001
IL-2/IFN-γ (≤ 47)	2.9	1.2–7.3	0.026
IP-10/IFN-γ (≤ 2330)	4.2	1.4–12.7	0.012
MIG/IFN-γ (≤ 285)	5.0	2.0–12.7	< 0.0006

OR, odds ratio; CI, confidence interval; *IU/ml.

## Discussion

In this study, we observed that the *Mtb*-specific T cell signature associated with ATB is characterized by a higher IFN-γ cell response, a significant CD8^+^ T cell response detectable in the second QTF-Plus tube, and a preferential IFN-γ production relative to IL-2, IP-10, and MIG secretions. IL-2, IP-10, and MIG responses to peptide antigen stimulation were assessed using ratios of cytokine productions measured by ELISA relative to IFN-γ secretion. We observed that IL-2, IP-10, and MIG indices were lower in the ATB group. Hence, low IL-2/IFN-γ, MIG/IFN-γ, and IP-10/IFN-γ ratios were associated with ATB with ORs ranging from 3 to 5. The poor secretion of MIG and IP-10 that are Th1-related cytokines induced by IFN-γ may reflect the impairment of the T cell response against TB (Collins and Kaufmann, 2001). These results were in line with previous studies highlighting that IL-2/IFN-γ secretion and a relative IL-2 toward IFN-γ production were determined in ATB compared to LTBI (Suter-Riniker et al., 2011; Jeong et al., 2015; Goyal et al., 2017; Suzukawa et al., 2020). Low secretion of IL-2 compared to IFN-γ production and high frequency of single positive tumor necrosis factor alpha (TNF-α) *Mtb*-specific CD4^+^ T cells have been associated with the defect in TB infection (Sargentini et al., 2009; Harari et al., 2011; Jenum et al., 2014). Hence, multicytokine *Mtb*-specific CD4^+^ T cell response is thought to protect against TB, while defects in cytokine may be the hallmark of ATB. Furthermore, IGRA based on other TB antigens, such as heparin-binding hemagglutinin antigen (HBHA), emerged as a promising tool for ATB diagnosis (Sali et al., 2018).

Although commercial IGRA is not intended to provide quantitative results, we previously reported that T-SPOT results over 100 IFN-γ secreting cells per 250,000 PBMC were more frequently observed in subjects with ATB (Carrère-Kremer et al., 2016). In this study, we confirmed that a strong IFN-γ response is more frequently detected in ATB than in LTBI in our patient population. We observed that QTF positive subjects with IFN-γ levels above 8 IU/ml were much more likely to have ATB compared to subjects with IFN-γ levels from 0.35 to 7.99 IU/ml (OR > 7) (Figure 3B,D,F). Our results were consistent with other studies performed in low burden TB countries,(Higuchi et al., 2008; Kobashi et al., 2010; Diel et al., 2011) suggesting that levels of IFN-γ response should be taken into account in the diagnostic workup of ATB in this setting. This benefit may be attenuated in high setting countries where strong T cell responses against TB are more frequently observed (Metcalfe et al., 2010; Ling et al., 2011).

In comparison with the prior versions of the assay, the QTF-Plus assay includes a second antigen tube stimulating IFN-γ production by both CD4^+^ plus CD8^+^ T cells. A stronger CD8^+^ T-cells response against ESAT-6 and CFP-10 antigens has been observed in subjects with active TB, high mycobacterial load, and recent exposure to TB compared to LTBI (Lancioni et al., 2012; Rozot et al., 2013; Allen et al., 2018). We observed a higher difference in IFN-γ production between the QFT-Plus tubes 1 and 2 in the ATB compared to LTBI. The median delta IFN-γ value between TB1 and TB2 exceeded 0.8 IU/ml in the ATB group, whereas a difference over 0.6 IU/ml has been considered as technically significant regarding the intrinsic variability of the test (Metcalfe et al., 2013; Barcellini et al., 2016; Lee et al., 2021). On the contrary, in the LTBI group, the level of IFN-γ released in TB1 and TB1 is similar, with a median difference close to zero between the two tubes, suggesting that the peptides designed to elicit immune responses from the CD8^+^ T cells marginally contribute to IFN-γ production in the second antigen tube.

Although the differences between ATB and LTBI groups were statistically significant, an important overlap between these groups was observed when we closely looked at their differences between the cytokine indices. Our studies were not dedicated to validating methods to distinguish ATB from LTBI in clinical practice. However, our results suggested that a better analysis of cytokine profile of T cell response against *Mtb* could provide valuable information about the equilibrium between host and pathogen and may be helpful to evaluate the risk of TB reactivation of ATB among IGRA positive patients.

In our two studies performed on IGRAs (T-SPOT and QuantiFERON), subjects were enrolled on a positive IGRA result. However, comparison between cytokine results using multiplex assay and ELISA is difficult because the two methods used different capture and reporter antibodies, as well as similar diluents and serum blockers, neither some quantity of biological compounds. In addition, while concordance is generally good when using cell supernatants, it is much less robust when using serum or plasma samples (Prabhakar et al., 2004).

Our study has several limitations which are as follows: (i) nine subjects only had extra-pulmonary TB; (ii) the number of subjects with ATB did not permit us to assess the possible association between TB localization, or delay from symptom onset, and cytokine patterns; (iii) we did not assess QTF response and cytokine patterns throughout TB treatment; (iv) we did not enroll ATB subject who tested negative for QTF when according to a recent meta-analysis, 8.6% (95 CI, 12.5–5.8%) of subjects with ATB tested negative for QTF Gold Plus assay (Oh et al., 2021); (v) our results cannot be extrapolated to the pediatric population (Basu Roy et al., 2020; Buonsenso et al., 2020).

In conclusion, giving attention to the level of IFN-γ response and TB1 vs. TB2 response, as well as analyzing cytokine response based on indices of IL-2, MIG, and IP-10 to IFN-γ in QFT supernatants, allowed to define profiles of T cell response that are more likely observed in ATB. In combination with other biological and clinical parameters, these approaches may be helpful to identify QTF-positive patients in whom microbiological tests and radiological examinations would be requested to rule out or confirm ATB.

## Data Availability Statement

The raw data supporting the conclusions of this article will be made available by the authors, without undue reservation.

## Ethics Statement

The studies involving human participants were reviewed and approved by the Sud-Méditerrannée-III, France, NCT02898623. The patients/participants provided their written informed consent to participate in this study.

## Author Contributions

SC-K, AP, KB, and ET: experimental work-up. MP, SG, and PK-D: data collection and statistical analyses. SC-K, PK-D, PV, ET, and AB: manuscript preparation and review. ET and SC-K: study design. All authors contributed to the article and approved the submitted version.

## Conflict of Interest

The authors declare that the research was conducted in the absence of any commercial or financial relationships that could be construed as a potential conflict of interest.

## Publisher’s Note

All claims expressed in this article are solely those of the authors and do not necessarily represent those of their affiliated organizations, or those of the publisher, the editors and the reviewers. Any product that may be evaluated in this article, or claim that may be made by its manufacturer, is not guaranteed or endorsed by the publisher.

## Abbreviations

ATB: active tuberculosis infection
AUC: areas under the curve
BCG: bacille Calmette-Guérin
IFN- γ: interferon-gamma
IL-2: interleukin-2
IGRA: interferon-gamma release assays
IP-10: IFN- γ inducible protein or CXCL-10
LTBI: latent tuberculosis Infection
MIG: monokine induced by IFN- γ or CXCL-9
MTB: *Mycobacterium tuberculosis*
QFT: QuantiFERON
TB: tuberculosis
TB1: Tube 1
TB2: Tube 2
TST: tuberculin skin test.
